# Systematic metabolite screening identifies orthosteric and allosteric regulators of the adenosine A2A receptor

**DOI:** 10.1063/4.0000864

**Published:** 2025-10-27

**Authors:** Prashant Rao, Manoj Rathinaswamy, Michelle Chan, Kevin Hicks, Amirhossein Mafi, Qi Hao

**Affiliations:** 1 Calico, San Francisco, California, USA; 2 University of Utah, Salt Lake City, UT, USA

## Abstract

The adenosine A2A receptor (A2AR) is a Class A G protein-coupled receptor (GPCR) broadly expressed in metabolically active tissues where it regulates inflammation, glucose metabolism, and energy homeostasis. While the effects of small-molecule ligands and protein interactions with A2AR have been extensively studied, the regulatory influence of endogenous metabolites remains unexplored. To address this gap, we employed the Mass spectrometry Integrated with equilibrium Dialysis for the discovery of Allostery Systematically (MIDAS) platform to systematically screen a library of 381 human metabolites, identifying over 100 that bind to A2AR, including prostaglandin D2 (PGD2), S-adenosylhomocysteine (SAH), and 2′-deoxyadenosine (DA). We discovered that the adenosine analogs - SAH and DA are orthosteric agonists of A2AR with EC50 values of 5 μM and 15 μM, respectively, using both protein and cell-based functional assays. In contrast, the anionic lipid PGD2, emerged as a weak negative allosteric modulator with an IC50 of 150 μM. To elucidate its binding site, we collected two single-particle cryo-EM datasets of A2AR in the presence and absence of PGD2, generating a difference map that revealed density features localized to the extracellular leaflet. Structure- activity relationship analysis of PGD2 analogs (PGD2-me, PGF2α, and PGK2) indicated that the presence and orientation of a single carbonyl group on the cyclopentane ring significantly enhanced inhibitory potency, whereas substituting it with a hydroxyl group reduced potency by two orders of magnitude. Furthermore, we employed MOE to visualize surface interactions likely to bind each functional group, which revealed preferential engagement of the carbonyl group to A2AR. Overall, our integrated strategy – combining cryo-EM, molecular docking, and biophysical assays supports a model in which PGD2 engages multiple binding sites to exert a cumulative inhibitory effect and provides a framework for exploring the mechanisms of weakly potent modulators.

